# Wound Care in Buruli Ulcer Disease in Ghana and Benin

**DOI:** 10.4269/ajtmh.13-0255

**Published:** 2014-08-06

**Authors:** Kristien Velding, Sandor-Adrian Klis, Kabiru M. Abass, Wilson Tuah, Ymkje Stienstra, Tjip van der Werf

**Affiliations:** Department of Internal Medicine, Infectious Diseases Service, University Medical Center Groningen and University of Groningen, The Netherlands

## Abstract

Buruli ulcer (BU) is a disease affecting the skin, subcutaneous fat, and bone tissues. Wound care is important in the prevention of disabilities. Awareness of current wound care practices in BU-endemic regions is necessary for future wound care interventions. Thirty-one health care workers in Ghana and Benin were interviewed with a semi-structured interview, complemented by structural observations. Quantitative data were analyzed through *t* tests and one-way analysis of variance, and qualitative data through descriptive statistics. There appeared to be a general understanding of wound assessment. A large variety of different topical antiseptics was reported to be used, pressure irrigation was never reported. Gauze was the main dressing type and a moist environment was preferred, but could not be maintained. Bleeding and pain were observed frequently. Standard of wound care differed importantly between health care personnel and between institutions and adherence to World Health Organization guidelines was low.

## Introduction

Buruli ulcer (BU) is an infectious skin disease caused by *Mycobacterium ulcerans*. BU has been reported in over 30 tropical countries worldwide, with the highest prevalence and incidence in West Africa.^1^ In most cases, BU starts as a small, painless swelling below the skin that may break down to form an ulcer. The current treatment consists of 8 weeks of oral rifampicin combined with intramuscular streptomycin, with healing rates of over 90%. Although antibiotics are successful in the treatment of BU, the reported median time to healing of ulcers was found to be 18 weeks in early, limited BU lesions^2^; the median time to healing for larger ulcers and those that require surgical intervention is unknown and likely to be longer. In addition, persistent wounds were found to be a risk factor for functional limitations.^3^,^4^ Combined with the long healing time, this implies that wound care is an important component of BU disease management and a major burden on the total costs of treatment.

Though solid evidence is lacking, it is likely that good wound care such as the choice of the correct type of dressing and a rational approach in applying topical solutions reduces time to healing, pain, and morbidity.^5^,^6^ For example, infection rates with the use of traditional gauze dressings were found to be significantly higher compared with moisture-retentive dressings in wounds of various origins.^5^,^7^ Modern dressing materials (e.g., hydrocolloids) have been shown to decrease time to healing and increased patient comfort.^5^,^8^–^10^

Basic principles of wound management are to treat or manage relevant systemic conditions, to maintain a moist wound environment,^11^ to protect the wound from trauma, to promote a clean wound base and control infection, and to control edema and lymphoedema.^12^ Currently, there are two guidelines on BU wound care, both published by the World Health Organization (WHO), which apply the basic principles of general wound care to BU.^12^,^13^ The goal of these guidelines is to minimize time to healing of wounds, optimize treatment outcome, and minimize iatrogenic damage. In these guidelines, wound care is described in a step-by-step manner: assessment of the wound, preparation of the wound bed, and dressing of the wound. Assessment of the wound is based on the “Red-Yellow-Black system” (RYB-system), as described in the WHO prevention of disabilities' manual.^13^,^14^ This is a simple method developed to classify a wound. The RYB-system classifies wounds by color (Table 1) and provides information on the phase of healing and basic principles of care required for each stage.

The second step in wound care is the preparation of the wound bed, for which WHO guidelines describe three steps. First, wash the wound and the surrounding area with water. Second, cleanse the wound with a normal saline solution (0.9% sodium chloride) by using low pressure irrigation. A simple and affordable method is to use a plastic bottle with a needle hole; the bottle is squeezed to gently spray the wound surface. Antiseptics should be avoided, as these can cause iatrogenic damage to newly forming epithelial and fibroblast cells.^15^–^17^ The use of antiseptics is restricted to highly infected wounds where the bacterial overgrowth is the major concern rather than the healing process. Finally, some yellow and black wounds need debridement. Debridement is ideally performed selectively, removing maximum amounts of dead tissue but minimizing damage to healthy tissue. Bleeding and severe pain are signs of damage to healthy tissues.

The type of dressing to be used is determined by the amount of exudate. Again, the color of the wound can be a helpful tool. Red wounds are often non-exudating. For these wounds, Vaseline gauze is recommended. In case of an exudating yellow or black wound a more absorbent dressing is advised. There are many types of absorbent wound dressings, but in countries with limited resources normal saline soaked gauze is often used because this is the cheapest form of dressing.

Little is known about the application of the basic principles of BU wound care as described by the WHO guidelines. This study aims to report on the current standard of BU wound care in Ghana and Benin. More specifically, information on wound assessment, dressing procedures, dressing materials, topical solutions, and resources available are reported for different hospitals and health care centers (HCCs) in Ghana and Benin. The information is gathered through semi-structured interviews, and is confirmed and complemented through observations in dressing rooms.

## Methods

### Participants.

A total of six hospitals and eight HCCs were visited between November 2010 and February 2011. In Benin two hospitals and three HCCs were approached. In Ghana four hospitals and five HCCs were included in the visit. All hospitals and HCCs visited in Ghana were localized in the Ashanti Region. This is the region with the highest prevalence and incidence of BU in Ghana. Together, these hospitals receive ∼50% of all reported BU cases in both countries. The number of cases received annually by each hospital did not differ greatly between the hospitals. The hospitals were not randomly selected, but rather approached through contacts at the regional and national level. All the HCCs visited were identified by hospital staff as their main BU wound care center for outpatients. Visits were announced to those in charge of the BU program at least 2 days before arrival. All health workers that were present and involved in BU wound care at the day of the visit were invited by the research team to participate in the study. There were no health care workers who declined to participate.

### Questionnaire.

**Figure 1.**
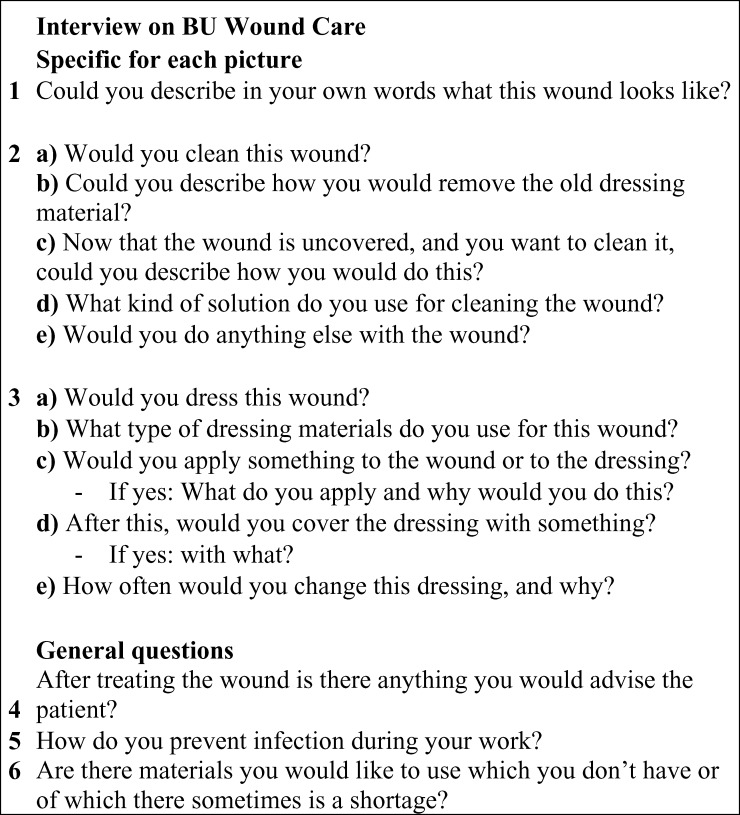
The interview.

Participants were shown six pictures of BU wounds, and were asked the same set of questions for each picture. In addition, every participant was asked three general questions. The questionnaire is shown in Figure 1, and is based on the WHO guideline for prevention of disabilities in BU and general principles of wound care.^13^ Participants were given the following introduction: “Next you will be shown six pictures of clinically confirmed BU cases. After showing you each picture I will ask you some questions on wound care of the BU cases depicted. Please, try to imagine that you are the one taking care of this patients' wound. This is not a test; there is no right or wrong answers. The goal of this research is to find out how wound care of BU is performed in endemic areas”.

**Figure 2.**
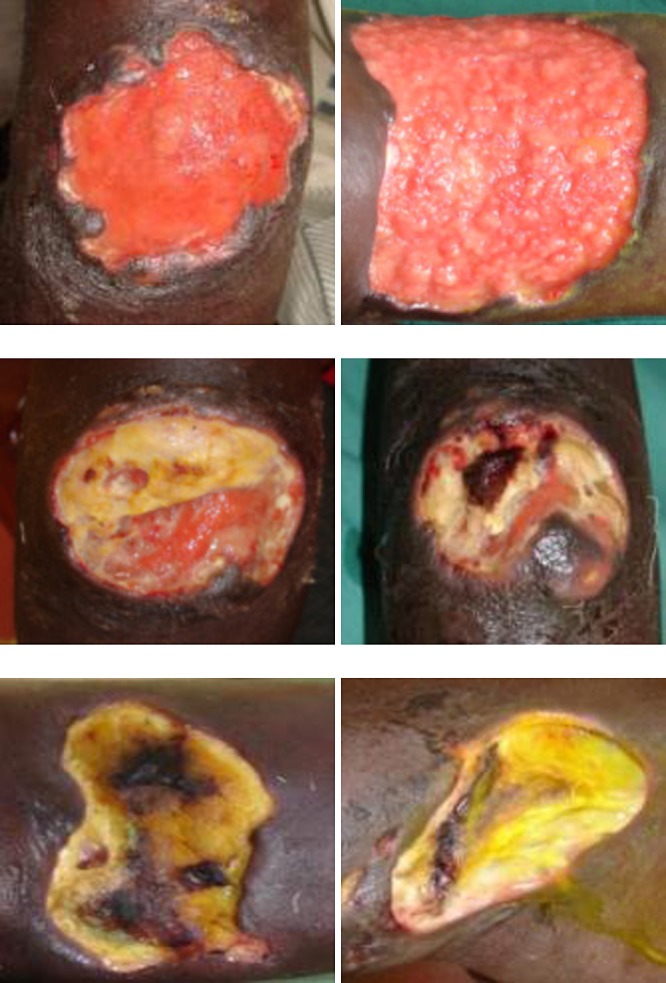
Pictures used in the Buruli ulcer (BU) wound care study. Top row: red wounds. Middle row: yellow wounds. Bottom row: black wounds.

Two of six pictures were classified beforehand by the study team as red wounds, two as yellow wounds, and two as black wounds. The pictures were shown in random order. Figure 2 shows the six pictures that were used.

### Observations.

Observations of the dressing change procedures at each facility were done to complement the information acquired by questionnaires. In contrast to the interviews, the observations concerned the procedures followed and materials used in the facility as a whole, rather than focusing on individual health care workers, although 25 of the 31 participants that were interviewed were also observed. The remaining six participants were not observed because no wound care was performed at the days of our visit. Observations were done before or after the interviews, depending on the clinical routine of the hospital or health center visited.

### Statistical analysis.

A sum score of 0–6 with one point for every picture that was described according to the WHO RYB guideline was calculated. Means of these scores were compared between male and female participants, between hospitals, between health care centers and hospitals, between occupations, and between countries. These comparisons were done by *t* tests and one-way ANOVAs. Significant differences in ANOVAs were further analyzed with Tukey and least significant difference (LSD) *post hoc* tests.

## Results

The hospitals and HCCs that were visited, with the number of subjects participating in the study in each hospital and associated HCCs are shown in Table 2. A summary of the aspects of wound care that were measured are listed in Table 3 .

### Participants.

Thirty-one interviews were conducted, and 68% of the participants were female. Fifty-eight percent were nurses by profession, 12% were nurses in training, and 30% had another occupation (e.g., ward assistant, community health worker).

### Wound assessment.

A total of 186 cases (six pictures for each of the 31 participants) were analyzed. The color of the wound was described correctly in 26% of all 186 cases. Red wounds were classified correctly in 35% of cases, yellow wounds in 24%, and black wounds in 19%. These differences in classification were not significant. Correct classification of five or six cases presented was achieved by 10% of all participants. Classification according to the guidelines did not differ significantly between gender and occupation of the health care worker, country, or level of the health care system.

Wound classification according to the WHO guideline differed significantly between the hospitals (*P* = 0.002). *Post hoc* tests (Tukey, LSD) showed that this statistically significant difference was caused by three of the six hospitals. These three were the hospitals with the highest rate of WHO guideline classification and two hospitals with the lowest rate of WHO guideline classification.

In addition to the color classification, wounds were often described by other characteristics. For example, red wounds were often described as clean wounds, or sometimes fresh wounds: “Clean and well-granulated wound, ready for a skin graft.” Black and yellow wounds were described as dirty and infected: “This is an infected wound, the color yellow, is not good,” “it is not clean.” Sometimes, participants commented on the duration of the disease, and on patient compliance (black wound): “This wound has been there for a long time,” “no compliance, the patient did not come for dressing,” “it is infected,” “there is no medical attention.” Or: “This one has not been daily dressed, I can tell because of the pus.” And for a red wound: “The redness of the wound says that it is healing and that the person is on drugs.” Only rarely were descriptions given of the edges, and the size: “Category II wound, this is a typical BU wound with undermined edges.” And: “It is 10 cm long, 6 cm high, and the middle is necrotic, I see a lot of fibrin and dry edges.”

### Preparation of the wound bed.

A common response when asked about removing the dressing and cleansing the wound was: “If old dressing material is adhered to the wound we use normal saline or tap water to remove it, in order not to cause damage to the wound or pain,” “I clean inside to outside and afterward the surroundings.” Forty-eight percent reported to remove the old dressing without moistening it. These results did not differ significantly for differently colored wounds. Washing of the wound and surroundings with water was reported to be done in two hospitals. These hospitals had washing facilities inside the dressing rooms, where patients were washed by staff or were to wash their wounds themselves. All participants reported that they would cleanse the wound. However, low pressure irrigation was never reported, or observed. The cleansing solutions used are listed in Table 3.

Forty-two percent of participants indicated that they would perform mechanical debridement in at least one of the six cases presented to them. This was confirmed by observations; mechanical debridement was performed in the dressing room either by cutting necrotic tissue with scissors of forceps. Common responses of participants were: “I will try to take some of the slough but I will not force it,” and “if the patient doesn't have too much pain we can take some of the necrotic tissue with the scissors.” In 2% of all red wounds presented the participants indicated to perform debridement, compared with 33% of all yellow wounds and 31% of all black wounds. Bleeding and severe pain were frequently observed during debridement procedures.

### Dressing of the wound.

Dressing materials did not differ among the hospitals and HCCs, only gauze and cotton wool was available for dressing. Participants frequently reported that they would soak the dressing with various solutions. The different solutions used are listed in Table 3. The solution most frequently reported to be used was normal saline, but the relative frequencies of the topical solutions used differed with the color of the wound. In practice, the differences in the use of solution for dressings depended on hospital policy. In most of the dressing rooms one other type of solution was available in addition to normal saline, and it was the availability that determined the choice of therapy. The solution used was the same for all BU patients independent of the characteristics of the patient or the wound. The frequency of dressings is listed in Table 3. Participants most often reported that they would change the dressing on a daily basis, and our observations confirmed this.

In four hospitals there was a dressing room available, at the other two hospitals wounds were dressed on the ward beds. Dressing rooms differed considerably in terms of size of the room and facilities (e.g., washing bay, running water, instruments). None of the HCCs had a dedicated dressing room available, and dressings were done in a random room or in the hallway instead. Most of the rooms used for dressing in the HCCs did not have running water or electricity.

### Prevention of infection.

A typical response was: “I wear new gloves for every patient,” “I wash my hands before every new patient and dry them with a clean towel,” “we use sterilized gauze,” and “we dress the BU patients in a different place than the other patients.” Ninety percent of the respondents indicated that they use gloves during wound care, although some used the same gloves for several patients. Gloves were available in the hospitals; however, HCCs were less well supplied. In one HCC the participant reported that one pair of gloves was used for all BU patients. A forceps to handle the dressing materials was reported to be used by 39% of respondents. Hand alcohol was indicated to be applied after every dressing by 13% of respondents, and 38% of respondents indicated that they washed their hands before dressing. Fifty-five percent indicated that they used masks, although several respondents said that it was to reduce the smell: “I use a face mask for highly infected wounds that smell badly.” Furthermore, wearing an apron, using sterilized instruments, hairnets, overshoes, and cutting the fingernails were answers given to this question by < 20% of the participants. Indeed, during our observations, all health workers used gloves. Forceps were often used, but these were non-sterile, and were usually used for multiple patients. In most hospitals, health workers wore aprons, either disposable or reusable, and roughly half of the health workers wore masks.

### Advice to the patient.

When asked what advice they would give the patient after dressing, 68% of respondents gave advice regarding wound care, e.g., to keep the wound dry, to apply Vaseline or cocoa butter to the wound. Fifty-two percent mentioned aspects of good hygiene, e.g., instructions to wash properly, at least once a day, and to clean the area around the wound. Sixty-one percent of the respondents gave some form of dietary advice, e.g., to eat more than normal, to eat more proteins like beans, groundnuts, and fish, and to eat more fruits or take vitamin C. Twenty-nine percent gave advice regarding exercise, e.g., physiotherapy, walking, although in contrast, two participants (6.5%) advised against exercise.

### Shortages.

An occasional or general lack or shortage in wound care materials was indicated by 71% of all participants. Most often a lack of sterile dressing materials was reported (36%) followed by a shortage in sterilized instruments (29%). Two participants at the same institution reported that there was no working autoclave (sterilizing equipment) available (6.5%). Other shortages that were reported by the participants were: items for personal protection (e.g., gloves, masks), running water, electricity, modern dressings, and topical solutions. Our observations confirm these findings, and when sterilized materials and equipment were unavailable, unsterilized dressing materials and instruments were used.

## Discussion

This is the first study that reports on BU wound care in endemic regions. We found that classification of BU wounds based on the RYB-system was not routinely done. However, there appeared to be a notion of clean healthy wounds (red wounds) versus dirty infected wounds (yellow and black wounds). Descriptions of the shape, size, and edges of the wound were rare. Classifying and monitoring wounds makes it possible to evaluate the effectiveness of the current treatment, and facilitates communication between nursing staff.^18^ Future wound care interventions may want to focus on the introduction of a simple nursing tool that provides caregivers with a daily assessment of the wound. Daily assessments should at least involve a description of the wound size, the edges, and the color.

Our results showed that washing of the wound and the healthy skin around the lesion was not a common procedure. Many BU patients work and live in the countryside and expose their extremities to dirt and dust, therefore it seems appropriate to wash the affected body area before cleansing or dressing. In one hospital, patients washed their wounds themselves. A potential benefit of this strategy is that patients are more involved in their own healing process, which could give them a sense of control, which in turn can reduce negative emotions and pain and have a positive effect on treatment outcome.^19^,^20^ Indeed, self care programs have been established before in leprosy patients, and these programs appeared successful in improving wound management.^21^

Nearly half of the respondents indicated that they would remove the old dressing without moistening it. The idea behind dry removal is that debris from the wound adheres to the gauze, and that removing the gauze provides some form of mechanical debridement. However, this technique is non-selective, i.e., it also removes newly granulating tissue with viable epithelium. Although little evidence exists, some consider it best to favor healing over debridement, and this is also recommended by the WHO for BU wound care.^13^,^22^ Participants reported to use scissors or forceps and cut dead or fibrinous tissue from the wound in about one-third of all yellow and black wounds presented. From our observations it became clear that debridement was often experienced as highly painful by the patient and severe bleeding was not uncommon. This indicates that the procedure is performed unselectively, and also healthy tissue instead of necrotic ones is removed. Thus, there seems to be a need for future training of health care workers on selective debridement techniques.

Cleansing by moderate-pressure irrigation of the wound with a saline solution, as described in the WHO guidelines, was not observed in any of the hospitals. Instead, cotton wool swabs or gauze was soaked in a solution and rubbed over the wound. However, moderate-pressure irrigation can be easily achieved by using a syringe, or making a hole in a water bottle, and is effective in reducing the bacterial load of a wound.^23^ Participants indicated to use normal saline for cleaning the wound in approximately half of the cases presented. Another solution that was frequently reported to be used for cleaning was hydrogen peroxide. Hydrogen peroxide is a debridement agent; it softens tissue and makes removal of slough or necrotic tissue easier. However, hydrogen peroxide was also reported to be used in red wounds. The use of hydrogen peroxide in red wounds delays wound healing and is contraindicated. Besides hydrogen peroxide, a large variety of different topical antiseptics were used to clean the wound. This is not surprising, as a large variety of topical antiseptics exist, and even in the most modern hospitals, choice of antiseptic and dressing material depends largely on the experience and preference of the nursing staff.^18^,^24^ In the institutions that we visited, the choice of cleaning solution depended largely on hospital policy and availability. However, it is best to reserve the use of antiseptics for cleansing to those wounds that show signs of infection. Although there is some debate, povidone-iodine is currently regarded as both safe and effective for this purpose.^23^,^25^ In addition, this solution is relatively inexpensive and widely available throughout Ghana and Benin.

Vaseline gauze was only reported to be used for dressing in about 4% of all cases, which was somewhat surprising. In red wounds, Vaseline gauze should be used because these wounds are not infected and have started healing, and the goal of care for a red wound is protection. As Vaseline gauze does not adhere to the wound surface, it minimizes pain and damage to healthy epithelial and fibroblast cells during dressing changes. Vaseline gauze is relatively inexpensive, and can be prepared by the hospital itself.

The frequency of dressing changes was reported to be once a day in the majority of cases, and from observations it became clear that this frequency was mainly determined by hospital policy rather than by wound characteristics. The only dressing material used was gauze, except for one hospital where staff sometimes used lipocolloids for dressing. Modern dressings such as hydrocolloids are superior in creating a moist wound bed, but are relatively expensive. Moist gauze, applied daily, seems like a reasonable alternative but a disadvantage of using moist gauze is that the moist environment is often not maintained. Under high temperatures initially wet gauze dries fast and acts as a foreign body, causing pain and bleeding during dressing changes. Therefore, the cost-effectiveness of modern dressings for BU should be studied, as their higher price might be offset by a reduction in staff costs as this dressing material can remain on the wound for several days. In addition, these dressings increase patient comfort, and some are occlusive and water resistant, which can be advantageous as many BU patients are farmers and are exposed to water and dirt frequently.

In many cases, unsterilized materials were used, or some of the sterilized materials were used for several patients. Obviously this carries a risk for infection, not only with wound flora, but also with blood borne viruses such as Hepatitis B virus and human immunodeficiency virus (HIV).

Our study suffers from several limitations. First, the health facilities were informed of our visits beforehand, which might have influenced the way the wound care was performed. However, as in any country, not informing the institutions of our visit would be considered to be impolite and inappropriate. Furthermore, some health workers were interviewed before the wound dressing was observed, for practical reasons this was unavoidable. The second limitation of this study was that the pictures used were not validated beforehand by experts other than the study team, and the “yellow” and “black” pictures are not completely yellow and black, but present a mixed picture. However, these cases were representative of clinical practice, where there is often a mixture of colors in the wound. In addition, we did not assess to what degree any of the health workers or health facilities had been exposed to the WHO guidelines but these guidelines are based on basic principles of wound care that nurses in Ghana and Benin are indeed exposed to during their education. Nonetheless, when conducting a future study the use of validated cases is recommended and it should be considered to train all staff on wound care beforehand.

In summary, BU wound care practices in Ghana and Benin differed from the WHO guidelines in several key aspects. The standard of wound care differed greatly on a personal level, and between institutions. Dressing facilities at HCCs often appeared less well equipped, where basic materials such as gloves and sterile gauze were not always available. Pressure irrigation was rare, a large variety of topical solutions was reported to be used and the use of Vaseline gauze uncommon. On average, red wounds were approached somewhat differently from black and yellow wounds, which are encouraging, but this was contrasted by several respondents that appeared to apply a “one size fits all approach.” Furthermore, a moist wound environment was often not maintained, wounds were exposed to trauma during dressing changes by removal of dry gauze from the wound bed and pain and bleeding were frequently observed during dressing procedures.

A high standard of wound care is likely to reduce the time to healing, decreasing the risk for secondary infections and functional limitations, and promoting an early return to society. Several low-cost interventions can be made to enhance the standard of wound care: using a simple system for classifying and monitoring the wound based on size and color, washing the area around the wound before dressing, using a syringe or pierced water bottle for low pressure irrigation of the wound, avoiding routine use of hydro-peroxide or chlorhexidine, and avoiding pain and bleeding. Furthermore, efforts should be made to empower health care workers responsible for wound care, e.g., with appropriate tools and facilities and further training in WHO guidelines on wound care management in BU patients.

## Figures and Tables

**Table 1:** Overview of the Red-Yellow-Black system as described by Krasner (1995)

	Surface appearance	Phase of healing	Care
Red	Pale pink to deep dark “beefy” red	Inflammatory phase	Clean with saline solution
			Cover to protect
			Keep wound bed moist
Yellow	Pale ivory, various shades of yellow, green, brown	Proliferation phase	Clean with saline solution
	Presence of “slough” (dead but moist tissue)		Debridement to reduce slough
	Generates much wound fluid (exudates)		Use absorbent wound covering
			Keep wound bed moist
Black	Black/brown or tan (thick, hard, and leathery)	Proliferation phase	Clean with saline solution
	Dead tissue that is dehydrated		Debridement of eschar
			Use absorbent wound covering
			Keep wound bed moist

**Table 3:** Reported wound care approach per wound type*

	Red	Yellow	Black	Total
Cleansing solutions				
Saline solution	77%	42%	37%	52%
Povidone iodine	13%	15%	18%	15%
Hydrogen peroxide	5%	32%	37%	25%
Other	5%	11%	8%	8%
Debridement				
Would consider debridement	2%	33%	31%	22%
Dressing				
Saline solution	45%	31%	34%	36%
Povidone iodine	18%	29%	24%	24%
Hydrogen peroxide	1%	10%	16%	9%
Metronidazole	16%	13%	13%	14%
Vaseline	7%	2%	3%	4%
Dry	7%	2%	0%	3%
Other	6%	14%	10%	10%
Frequency of dressings				
Twice daily	10%	8%	10%	9%
Daily	65%	92%	87%	81%
Several times per week	25%	0%	3%	9%

* Other = sodium hypochlorite, sugar, vinegar, ciprofloxacin.

**Table 2:** Hospitals and health posts visited*

Hospital	HCCS	Country	No. of interviews
Tepa Government Hospital	Mamfo, Anyinasuso	Ghana	6
Nkawie-Toase Government Hospital	Abuakwa	Ghana	5
St. Martins Catholic Hospital Agroyesum	Tontonkrum	Ghana	6
Agogo Presbyterian Hospital	Ananekrum	Ghana	6
CDTUB de Pobè	Anigbolo, Issaba	Benin	5
CDTUB de Lalo	Adoukandji	Benin	4

* CDTUB = Center de Dépistage et de Traitement de l'Ulcère de Buruli.
